# *Lactobacillus casei* Exerts Anti-Proliferative Effects Accompanied by Apoptotic Cell Death and Up-Regulation of TRAIL in Colon Carcinoma Cells

**DOI:** 10.1371/journal.pone.0147960

**Published:** 2016-02-05

**Authors:** Angeliki Tiptiri-Kourpeti, Katerina Spyridopoulou, Valentina Santarmaki, Georgios Aindelis, Evgenia Tompoulidou, Eleftheria E. Lamprianidou, Georgia Saxami, Petros Ypsilantis, Evangeli S. Lampri, Constantinos Simopoulos, Ioannis Kotsianidis, Alex Galanis, Yiannis Kourkoutas, Dimitra Dimitrellou, Katerina Chlichlia

**Affiliations:** 1 Department of Molecular Biology and Genetics, School of Health Sciences, Democritus University of Thrace, University Campus-Dragana, 68100 Alexandroupolis, Greece; 2 Department of Hematology, School of Health Sciences, Democritus University of Thrace, University Campus-Dragana, 68100 Alexandroupolis, Greece; 3 Laboratory of Experimental Surgery & Surgical Research, School of Health Sciences, Democritus University of Thrace, 68100 Alexandroupolis, Greece; 4 Department of Pathology, Medical School, University of Ioannina, Ioannina, Greece; Agricultural University of Athens, GREECE

## Abstract

Probiotic microorganisms such as lactic acid bacteria (LAB) exert a number of strain-specific health-promoting activities attributed to their immunomodulatory, anti-inflammatory and anti-carcinogenic properties. Despite recent attention, our understanding of the biological processes involved in the beneficial effects of LAB strains is still limited. To this end, the present study investigated the growth-inhibitory effects of *Lactobacillus casei* ATCC 393 against experimental colon cancer. Administration of live *Lactobacillus casei* (as well as bacterial components thereof) on murine (CT26) and human (HT29) colon carcinoma cell lines raised a significant concentration- and time-dependent anti-proliferative effect, determined by cell viability assays. Specifically, a dramatic decrease in viability of colon cancer cells co-incubated with 10^9^ CFU/mL *L*. *casei* for 24 hours was detected (78% for HT29 and 52% for CT26 cells). In addition, live *L*. *casei* induced apoptotic cell death in both cell lines as revealed by annexin V and propidium iodide staining. The significance of the *in vitro* anti-proliferative effects was further confirmed in an experimental tumor model. Oral daily administration of 10^9^ CFU live *L*. *casei* for 13 days significantly inhibited *in vivo* growth of colon carcinoma cells, resulting in approximately 80% reduction in tumor volume of treated mice. Tumor growth inhibition was accompanied by *L*. *casei*-driven up-regulation of the TNF-related apoptosis-inducing ligand TRAIL and down-regulation of Survivin. Taken together, these findings provide evidence for beneficial tumor-inhibitory, anti-proliferative and pro-apoptotic effects driven by this probiotic LAB strain.

## Introduction

Probiotics, such as lactic acid bacteria (LAB), are important microorganisms in a healthy human microbiotic environment. They have recently acquired considerable importance because of their health beneficial properties [1–5]. It is believed that they form a stable and resilient gut ecosystem enriched for species that exert a concerted beneficial effect on the host immune system [6–7]. Taken into consideration that probiotics colonize the intestine and can exert their effects in the local mucosal tissue, they are relevant for alleviating the pathology of intestinal disorders. In fact, dysbiosis of the microbiota in the gastrointestinal tract is considered to be one of the contributing factors in the development of certain gastrointestinal diseases and malignancies [8]. In particular, an imbalance of commensal bacteria and their gene products underlies gastrointestinal inflammation and a predisposition to cancer. Microflora dysbiosis occurs at the mucosal surface in colonic adenomas, and may represent a potential factor for dysplastic cell proliferation [9–10]. Colorectal cancer is regarded a complication of inflammatory bowel disease, and several studies show that chronic inflammation can predispose the intestinal epithelium to malignant transformation [11]. To this end, recent reports showed the potential of the administration of specific probiotic strains to improve the balance of the gastrointestinal microbiota altered in different diseases [3–5,12].

Probiotics may modulate the activities of the intestinal microflora via different mechanisms. They act through various molecular and cellular mechanisms that involve i) adhesion to pathogen bacteria, ii) enhancement of innate immunity, iii) decrease of pathogen-induced inflammation, and iv) promotion of various gut homeostasis, processes such as, intestinal epithelial cell survival, barrier function, and protective responses [3,13]. Some of these beneficial effects may be attributed to secreted probiotic-derived factors, therefore soluble metabolites. Such metabolites have recently been identified as "postbiotic" mediators [14–16].

Lactic acid bacteria, and especially, species of the genus *Lactobacillus*, have received considerable attention as examples of beneficial microbiota, exerting immunomodulatory, anti-inflammatory and anti-carcinogenic activities [1,2–5,12]. Their effects are dependent on the strain-specific metabolic properties, the molecules presented and the components secreted. Thus, the individual combination of such properties in a certain probiotic strain determines its specific probiotic action, and as a consequence, its effective application for the prevention and/or treatment of a certain disease [13]. Anti-proliferative effects of various *Lactobacillus* strains as well of components thereof against cancer cells were reported [17–21], and pro-apoptotic effects [21–23] as well as autophagic cell death [24] described.

A number of *in vivo* animal studies indicate that lactobacilli may alleviate the risk of certain types of cancer. It appears that lactobacilli exert anti-carcinogenic properties by altering the gastrointestinal microflora and colonic metabolism, degrading carcinogens, producing anti-mutagenic compounds and enhancing host’s immune responses [1,25–30]. Anti-tumor effects of lactobacilli have also been described [31–37]. Randomized clinical studies have raised the possibility that certain lactobacilli may be promising for colon cancer prevention [38–39]. Despite these findings, our understanding of the biological processes mediating strain-specific direct anti-neoplastic activities of LAB is still limited.

However, the exact role of defined strains and components thereof in experimental colon cancer models has not been widely explored. In this context, the present study investigated biological activities mediated by the LAB strain *Lactobacillus casei* ATCC 393, a key microorganism in fermented dairy products and foods [40–43]. Our results provide further evidence for growth-inhibitory, pro-apoptotic and anti-tumor effects of *Lactobacillus casei* against colon carcinoma *in vitro* and *in vivo*.

## Materials and Methods

### Cell lines

Human HT29 [44] and murine CT26 [45–46] colon carcinoma cell lines were maintained under sterile conditions at 37°C in a humidified atmosphere of 5% CO_2_, and routinely cultured in standard DMEM medium supplemented with 10% heat inactivated fetal bovine serum (FBS, Life Technologies), 2 mM glutamine and 100 u/mL penicillin / 100 μg/mL streptomycin (Life Technologies). For the co-incubation experiments with *L*. *casei*, cells were maintained at 37°C under CO_2_ independent conditions cultured with the above described standard DMEM medium supplemented with 25 mM HEPES.

### Bacterial culture conditions and preparation of *L*. *casei* extracts/fractions

*Lactobacillus casei* ATCC 393 (DSMZ, Germany) was grown in MRS Broth at 37°C without agitation. Bacteria were harvested in late-log/early stationary phase of growth (10^9^ CFU/mL) by centrifugation at 1700 g for 15 minutes at 4°C. After washing with sterile phosphate-buffered saline (PBS), live *L*. *casei* was adjusted to the appropriate density in DMEM medium (for the *in vitro* experiments) or saline solution (for the *in vivo* experiments). The number of lactobacilli (CFU *L*. *casei* / mL) was determined by serial dilution and plating on acidified MRS agar

For soluble *L*. *casei* fractions, *L*. *casei* bacteria were cultured overnight, and cell density was adjusted to 10^9^ CFU/mL. For the production of cell-free supernatant (CFS), an overnight (late log/early stationary phase) *L*. *casei* culture was centrifuged (1700 g, 15 minutes, 4°C) twice and passed through a 0.22 μm filter. For the production of the heat-killed sonicated (HK-SON) fraction, an overnight *L*. *casei* culture was heated at 100°C for 40 minutes, while stirring it every 10 min. Heat-killed *L*. *casei* bacteria were then sonicated (10 rounds, 1 minute/ round, 70% amplitude, 50W) and centrifuged (13000 g, 40 minutes, 4°C). Protein concentration of soluble *L*. *casei* fractions/extracts was determined using the BCA protein assay kit (Thermo Scientific) according to the manufacturer’s instructions. Briefly, 100 μl of sample was added to 200 μL of the reagent mix following incubation at 37°C for 30 minutes. Samples were cooled down to room temperature and absorbance was measured at 562 nm on a microplate reader (Tecan). Serial dilutions of BSA were used to create an absorption-concentration standard curve. The concentration for CFS was approximately 10 mg protein/mL and for the HK-SON fraction 15 mg/mL.

### Cell viability assays

Cell viability was determined using the SRB assay [47] for CT26 and HT29 cells at an initial cell density of 5,000 or 20,000 cells per well, respectively. Cells were incubated with live *L*. *casei* or soluble extracts for 24, 48 and 72 hours. Cells were washed with PBS and fixed with 10% TCA. Then, cells were stained with SRB for 30 minutes and repeatedly washed with 1% acetic acid as previously described [47]. The dye was dissolved in 10 mM Tris base, and absorbance was determined at 492 nm using a microplate reader (Tecan). Cells treated with PBS or MRS served as controls.

### Effect of pH on cell growth

The effect of the pH of culture medium on CT26 and HT29 cell growth was determined using the SRB assay. After seeding, cells were cultured on 96 well plates for 24 or 48 hours. Culture medium was substituted with HEPES-supplemented DMEM, and pH was adjusted to values ranging from 6.0 to 7.0. Following incubation for 24 hours under CO_2_ independent conditions, SRB was performed as described above.

### HPLC analysis

The concentrations of short-chain fatty acids (SCFA), such as lactic, acetic, propionic and butyric acid, as well as ethanol and glucose, present in supernatants of cells co-incubated with *L*. *casei*, were determined by high-performance liquid chromatography (HPLC). Prior to analysis, 4 ml of each sample were mixed with 150 μL of 85% trichloroacetic acid (TCA) and then placed in an ice bath for 45 min. Subsequently, the samples were centrifuged at 19,000 g for 20 min. Prior to analysis, the supernatants were passed through 0.22 μm filter membranes. The HPLC system consisted of a Shimadzu chromatograph with a NUCLEOGEL 300 OA, 300x7.8mm ion exchange column (Macherey-Nagel, USA), coupled with a guard column, an LC-20AD pump (Shimadzu, Germany) and a CTO-20AC oven (Shimadzu) operated at 55°C. The target compounds were detected using a RID-10A refractive index detector. A sample of 40 μL was injected into the column and 0.01 N sulphuric acid was used for elution at a flow rate of 0.4 mL/min. The concentrations of glucose, organic acids and ethanol were estimated based on standard curves.

### Determination of bacterial cell adhesion by live confocal microscopy and flow cytometry

For the detection of bacterial cell adhesion by live confocal fluorescence microscopy, *L*. *casei* ATCC 393 or *E*. *coli* ATCC 25922 were labeled with 20 μM CFSE (CellTrace CFSE Cell Proliferation kit, Invitrogen). CT26 or HT29 cells were seeded on 8-well μ-slide (IBIDI) and grown for 24 hours until they reached 60–80% confluency. Cells were treated with 10^9^ CFU/mL *L*. *casei* or *E*. *coli* for 2–5 hours. Nuclei were detected by Hoechst staining and cytoplasmic membranes were stained with CellBrite Red (Biotium). Bacterial adhesion was visualized on a confocal microscope. For the detection of bacterial cell adhesion by flow cytometry, a modified protocol based on the method of Bianchi *et al*. [48] and Kodaikkal *et al*. [49] was developed to visualize the adhesive properties of lactobacilli. Briefly, cells were seeded in 10 cm^2^ culture plates and left to adhere overnight. Live *L*. *casei* bacteria were labeled with 20 μM CFSE. Cells were incubated with 10^8^ CFU/ml of fluorescently-labeled *L*. *casei* for 24 hours or with 10^9^ CFU/mL labeled bacteria for 2 hours. Following the incubation period, culture medium was discarded and cells were thoroughly washed with PBS to remove non-adherent bacteria. After trypsinization, cells were washed with PBS twice and analyzed on a flow cytometer (Calibur, BD Biosciences) for the detection of CFSE positive events and FL1 intensity. Non-treated cells and labeled bacteria, serving as negative and positive controls respectively, were also analyzed.

### Determination of apoptosis by Annexin V and Propidium Iodide staining

Apoptosis was assayed using the Annexin V—Propidium iodide (PI) method [50–51] with a commercially available kit (BD Biosciences) according to the manufacturer’s instructions. CT26 or HT29 cells were seeded in 10 cm^2^ cell culture plates at a density of 2x10^6^ cells per plate and left to adhere overnight. Upon reaching 70–80% confluency, cells were treated with 10^8^ or 10^9^ CFU/mL *L*. *casei* for 24 hours. Following treatment, cells were trypsinized, washed thoroughly and resuspended in Annexin binding buffer according to the manufacturer’s protocol. Cells were labeled by adding Annexin V-FITC and PI in each sample. After incubation for 15 minutes at room temperature in the dark, samples were analyzed on a flow cytometer (Calibur, BD Biosciences) for the detection of Annexin V and PI positive subpopulations. Non-treated cells were used as control. Further analysis was performed with FlowJo V10 software. Moreover, *L*. *casei*-treated Annexin V and/or Propidium iodide positive cells were visualized on a confocal microscope. Apoptotic nuclei were also visualized on a confocal microscope using 10 μg/mL Hoechst 33342 stain. For the respective experiments, HT29 or CT26 cells were grown on 8-well μ-slide (IBIDI) and treated with 10^9^ CFU/mL *L*. *casei* for 24 hours.

### Imaging by confocal microscopy

Imaging was performed on a customized Andor Revolution Spinning Disc Confocal System built around a stand (IX81; Olympus) with the 60x and 100x 1.4 NA lens and a digital camera (Andor Nixon+885) (CIBIT Facility, MBG-DUTH). Optical sections were recorded every 0.5–1 um. Image acquisition was performed in Andor IQ2 software. Z-stack images are two dimensional maximum intensity projections.

### Gene expression by Real-time PCR analysis

RNA was extracted from cells using the Trizol reagent (Life Technologies). The quality and concentration of RNA were examined by ethidium bromide-stained agarose gel electrophoresis and spectrophotometric analysis. cDNA template was synthesized from 1 μg RNA with the PrimeScript cDNA synthesis kit (Takara). The real-time PCR reaction mixture consisted of a 10 μL total volume solution containing 5 μL of 2x KAPA SYBR^@^ FAST qPCR Master Mix ABI Prism, 0.2 μL of 10 μM of each primer, 10 ng cDNA template and PCR-grade water up to 10 μL. The reactions were carried out on a StepOne PCR System (Applied Biosystems) in MicroAmp® Fast Optical 48-Well Reaction Plates or MicroAmp™ Optical 8-Cap Strips using the KAPA SYBR Fast MasterMix ABI Prism (KAPA Biosystems) reagent. The PCR conditions were 95°C for 2 minutes followed by 40 cycles of 95°C for 2 seconds and 60°C for 30 seconds. RT-PCR primers (VBC Biotech) were designed using Primer3 software to have the same Tm (60°C) and were as follows; murine TRAIL forward primer: CCCTGCTTGCAGGTTAAGAG and reverse primer: GGCCTAAGGTCTTTCCATCC; murine cyclin D1 forward primer: AGTGCGTGCAGAAGGAGATT and reverse primer: CACAACTTCTCGGCAGTCAA; murine BIRC5a forward primer: GACCACCGCATCTC and reverse primer: AAGTCTGGCTCGTTC; murine beta-actin forward primer: CGGTTCCGATGCCCTGAGGCTCTT and reverse primer: CGTCACACTTCATGATGGAATTGA. Primer specificity was verified by performing a melting curve analysis. Endogenous expression of beta actin was used as the internal reference. TRAIL, cyclin D1 and BIRC5a mRNA expression levels were evaluated by the comparative quantification Ct method (ΔΔCt) [52]. Statistical analysis was performed by SPSS 19 software. Normality was determined with the Kolmogorov-Smirnov test. Groups that passed normality test were analyzed with a Student's t-test, otherwise a Mann-Whitney test was used.

### Western Blot Analysis

CT26 or HT29 colon cancer cells were cultured on 10 cm^2^ cell culture dishes (Corning) and were treated with 10^8^ CFU/mL *L*. *casei* for different time points. Control cells were grown in DMEM HEPES-supplemented culture media in the absence of the probiotic strain for 24 hours. Cells were lysed using a homogenization buffer (250 mM sucrose, 1 mM EDTA, 10 mM Tris HCl buffer, pH 7.2 in the presence of protease inhibitors) and sonicated (slightly modified from the protocol described by Hitchcock M (http://www.unr.edu/proteomics/sample-preparation/2d-protocols/protocol-protein-fractions)), in order to separate and retrieve the membrane and soluble protein fraction. Protein concentrations were determined using the BCA protein assay kit (Thermo Scientific). TRAIL protein was detected by a standard Western blot analysis. Briefly, equal amounts of the protein extracts were loaded onto a 12% SDS polyacrylamide gel (Bio-Rad), electrophoresed and blotted onto a PVDF membrane (0.2 μm, Immobilon, Millipore). The membrane was blocked using 5% (w/v) dry milk and then probed overnight at 4°C with primary antibodies. A rabbit anti-human/mouse antibody was used to detect TRAIL at approximately 32 kDa (1:50 dilution, AbCAM) followed by incubation with a HRP-conjugated anti-rabbit secondary antibody. The protein band was visualized by autoradiography using ECL HRP chemiluminescent substrate (Life Technologies) and exposing to Kodak film. As loading control, an anti-β-tubulin antibody (1:5000 dilution, Sigma-Aldrich) was used and detected at about 55 kDa with an HRP-conjugated anti-mouse secondary antibody. A pre-stained protein marker was used to monitor protein molecular weight (Nippon Genetics).

### TRAIL detection by immunofluorescence

Colon cancer cells were grown on glass coverslips on 10 cm^2^ cell culture dishes (Corning) and were treated with 10^8^ CFU/mL *L*. *casei* for 12 hours or 10^9^ CFU/mL for 5 hours. Cells were fixed and permeabilized with ice-cold methanol for 5 minutes at -20°C, washed with PBS and blocked with 3% BSA in PBS, following incubation for 2 hours with a rabbit anti-mouse/human primary TRAIL antibody (1:200 dilution, Abcam). Cells were washed and blocked again, and incubated with an anti-rabbit AlexaFluor647-conjugated secondary antibody (Biotium). Coverslips were mounted with Mowiol and imaged by confocal fluorescence microscopy.

### Immunohistochemical Analysis

Tumors were fixed in 10% formalin and then dehydrated in graded concentrations of ethanol, xylole and finally embedded in paraffin. Serial sections 3 μm thick were prepared from the formalin-fixed, paraffin-embedded tissue blocks and floated onto charged glass slides. A hematoxylin and eosin stained section was obtained from each tissue block. Immunostaining was performed on formalin-fixed, paraffin-embedded tissue sections by the streptavidin-biotin peroxidase labeled method. All sections were deparaffinized and hydrated using graded concentrations of ethanol to deionized water. Tissue sections were subjected to quenching of endogenous peroxidase and antigen retrieval using microwaving in high pH citrate buffer. The primary antibodies were then applied to the tissues at a dilution 1:50 (overnight incubation at 4°C). Bound antibody was then visualized with DAB chromogen, followed by counterstaining with hematoxylin. Tissue sections incubated only with secondary antibody served as negative controls. An image analysis system composed of the Olympus BX43 upright microscope, digital camera Olympus Cam-SC30 and soft analysis (analySISH) was used in the tumor sections (stained with antibodies and counterstained with hematoxylin). Immunostaining was assessed by a developed continuous score system. The number of immunopositive cells was divided by the total number of the counted cells, and the expression was defined as the percentage of positive cells in the total number of counted cells.

### Animals

BALB/c mice were raised in the Animal House unit (Laboratory of Experimental Surgery and Surgical Research at Democritus University of Thrace, Nr. EL71 BIO2). A total of 40 female BALB/c mice (6–8 weeks old, weight 20–25 g) were used. They were housed in polycarbonate cages, max. 10 mice per cage, at room temperature, on a 12h light-12h dark cycle and were provided with tap water and commercial pelleted diet (Mucedola) free of lactobacilli *ad libitum* in sterile cages.

### Lactobacillus administration and syngeneic tumor model

The experimental protocol was approved by the Animal Care and Use Committee of the local Veterinary Service and was in compliance with Directive 86/609/EEC. Female BALB/c mice (6–8 weeks old, weight 20–25 g) were separated into two independent groups (10 mice per group). For a period of 13 days, live 10^9^ CFU *L*. *casei* suspended in PBS, were administered *per os* daily in a final volume of 150 μl. Mice in the control group received an equal volume of PBS. At day 10, 5x10^6^ CT26 cells per mouse were injected subcutaneously as a single dose, and seven days post CT26 inoculation, mice were euthanized by cervical dislocation and tumors were excised. Tumor volume and incidence were determined. Tumor dimensions were measured by an electronic micrometer and tumor volume was calculated using the modified ellipsoid formula (width^2^xlength)/2. During the course of the experiments the weight of each mouse was monitored, as well as signs of disease or discomfort. All efforts were made to ameliorate suffering. The experiment was repeated once to confirm the results.

### Statistical evaluation

Data were analyzed with SigmaPlot 11.0 and SPSS 19 software and statistical differences were analyzed by performing independent samples Student's t-test (normal distribution) or Wilcoxon signed-rank test (non-parametric) where appropriate. Differences between control and *Lactobacillus*-treated groups were considered significant when *p* <0.05. A minimum power of 0.80 was used when calculating sample size.

### Ethics statement

Animal experiments were approved by the Animal Care and Use Committee of the Veterinary Department of Evros Prefecture (license number 4766/28-3-2013) since it complied with the requirements set by Directive 86/609/EEC and PD 160/91 which was the legislation in force at the time of experimentation. All animal experiments were conducted in light of 3 R’s (replacement, refinement, reduction) and all mice used for the experiments were not subjected to pain or discomfort.

## Results

### *Lactobacillus casei* inhibits proliferation of colon cancer cells

**Fig 1:**
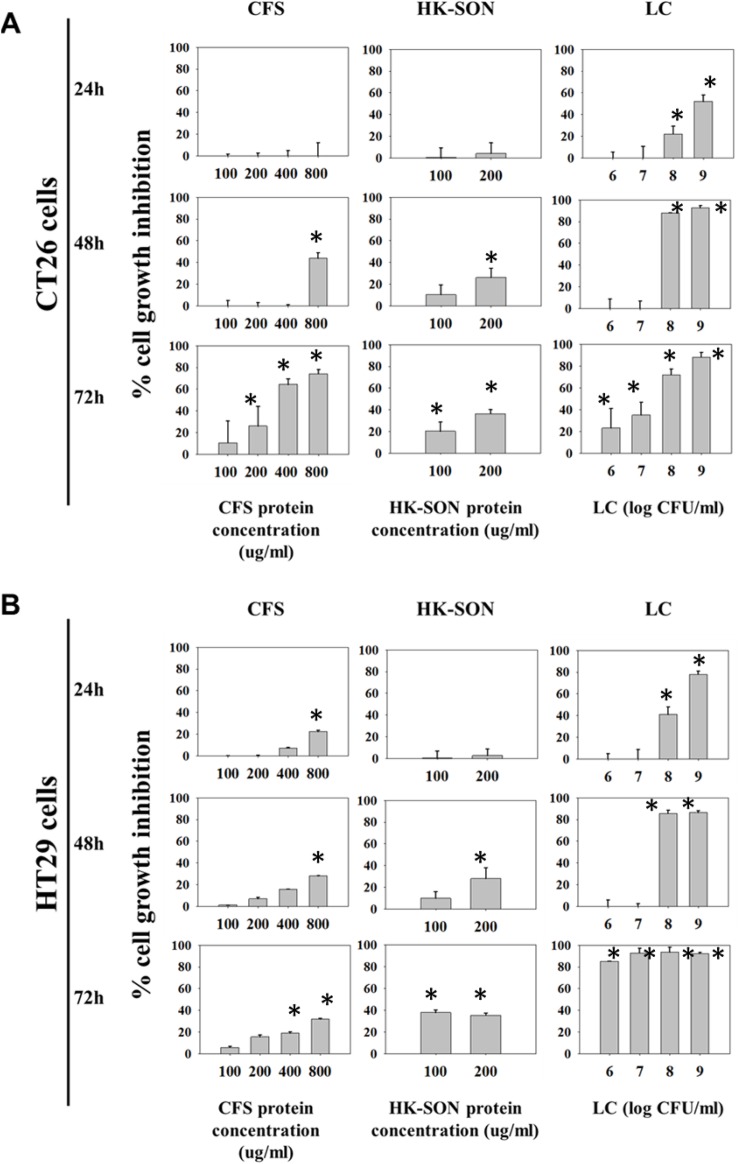
*Lactobacillus casei* inhibits proliferation of colon cancer cells. Anti-proliferative effect of increasing concentrations of selected preparations of *L*. *casei* cell-free-supernatant (CFS), heat-killed sonicated (HK-SON) and live *L*. *casei* (LC) at different time points on murine CT26 (A) and human HT29 (B) colon cancer cells, determined by the sulforhodamine B assay. Percentages of growth inhibition of *L*. *casei*-treated to control PBS-treated cells and CFS/HK-SON-treated to MRS-treated cells are presented as mean values ± *s*.*d*. from eight replicates. All data shown are representative of at least 4 independent experiments. Data were analyzed with the statistical software SPSS using Student’s *t*-test. Differences between control and treated groups are considered statistically significant when *p* < 0.05 (*).

The growth-inhibitory effect of *Lactobacillus casei* ATCC 393 was examined *in vitro* against human and mouse colon cancer cells. The percentage of growth inhibition of increasing concentrations of live *L*. *casei* (and CFS or HK-SON) against human HT29 and murine CT26 colon cancer cell lines is shown in Fig 1. Live *L*. *casei* as well as extracts thereof (secreted products present in CFS or bacterial components present in the HK-SON fraction) induce anti-proliferative effects *in vitro* and reduce the viability of murine (CT26) or human (HT29) colon cancer cell lines in a concentration- and time-dependent manner. Notably, the most pronounced anti-proliferative effect was induced by live *L*. *casei* on both cell lines. Indeed, co-incubation of CT26 or HT29 cells with 10^8^ or 10^9^ CFU/mL live *L*. *casei* for 48 hours almost completely inhibited cell growth. Even 24 hours following co-incubation, live *L*. *casei* significantly inhibited the growth of colon carcinoma cells. At concentrations of 10^9^ CFU/ml for 24 hours, a 52% reduction in cell growth was observed for CT26 (Fig 1A), while a 78% growth inhibition was determined for HT29 cells (Fig 1B). In parallel, the inhibitory effect was monitored by microscopic observation.

### Acidic pH in culture medium is only partly involved in the *Lactobacillus casei*-induced anti-proliferative effect

**Fig 2:**
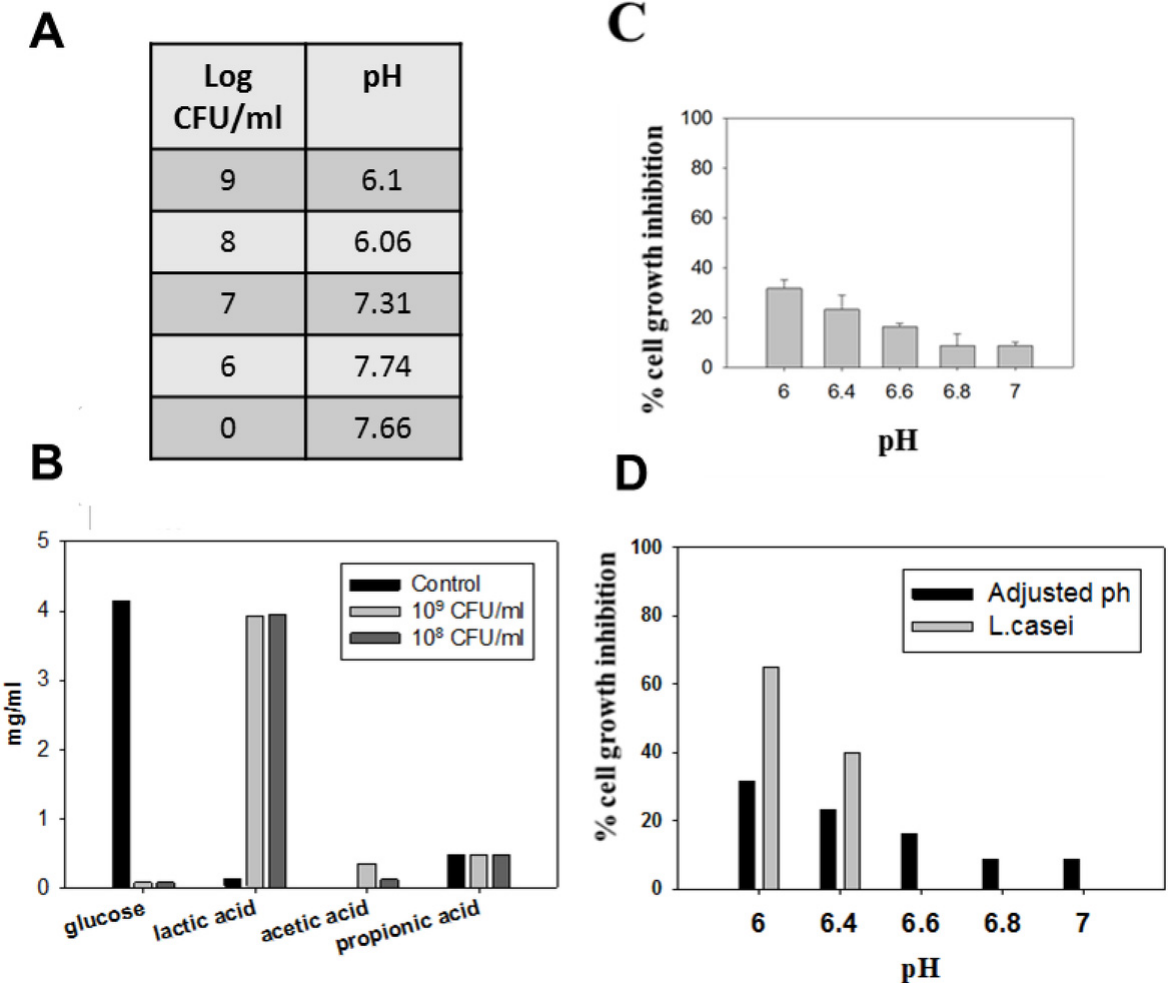
Acidic pH in culture medium is only partly involved in the *Lactobacillus casei*-induced anti-proliferative effect. (A) pH values measured in culture supernatants (see Materials and Methods) after co-incubation of CT26 cells with increasing concentrations of live *L*. *casei* for 24 hours. Control cells (indicated as 0) were cultured for the same time period in the absence of *L*. *casei*. (B) Lactic acid production in the supernatant of *L*. *casei*-treated CT26 cells after a 24 hour co-incubation period. Note the consumption of glucose. (C) Effect of acidic pH on the proliferation of CT26 cells. The percentage growth inhibition refers to control CT26 cells cultured in standard DMEM medium having a pH of 7.66 (D) Comparison of the growth-inhibitory effect of different concentrations of live *L*. *casei* on CT26 cells versus the effect of pH adjusted medium. Values of pH are comparable (see 2A) to those induced by co-incubation with higher concentrations of live bacteria. Results were reproduced in 3 independent experiments.

In order to investigate the correlation between the anti-proliferative effect of *L*. *casei* and pH decrease in cell culture medium due to bacterial acid production, we performed cell viability experiments culturing cells to a range of predefined pH values (6.0–7.0), selected to correspond to pH measurements of culture medium supernatants following co-incubation of CT26 cells with live *L*. *casei* (Fig 2A). As expected, co-incubation of cells with increasing doses of *L*. *casei* for 24 hours resulted in a dose-dependent pH decrease in culture medium (Fig 2A). HPLC analysis of the culture supernatant showed high lactic acid production. Apparently, glucose was almost completely consumed (Fig 2B). In order to test the effect of *L*. *casei*-induced acidic pH on cell proliferation, CT26 cells were cultured in DMEM with acidity adjusted to the selected pH values. A pH decrease below 7.0 significantly reduced the viability of CT26 cells; the inhibitory effect, as compared to control cells incubated in medium with a pH of 7.6 (Fig 2A), was proportional to the pH decrease (Fig 2C). We observed that acidic pH alone was not responsible for the anti-proliferative activity of *L*. *casei* against murine CT26 colon cancer cells, since, acidic pH values evoked lower growth-inhibitory effects than the co-incubation of cells for 24 hours with live bacteria (Fig 2D). Thus, for the time period indicated, part of the growth-inhibitory effect of *L*. *casei* is independent of bacteria-induced pH decrease.

### Fluorescently-labeled live *Lactobacillus casei* adheres to colon cancer cells *in vitro*

**Fig 3:**
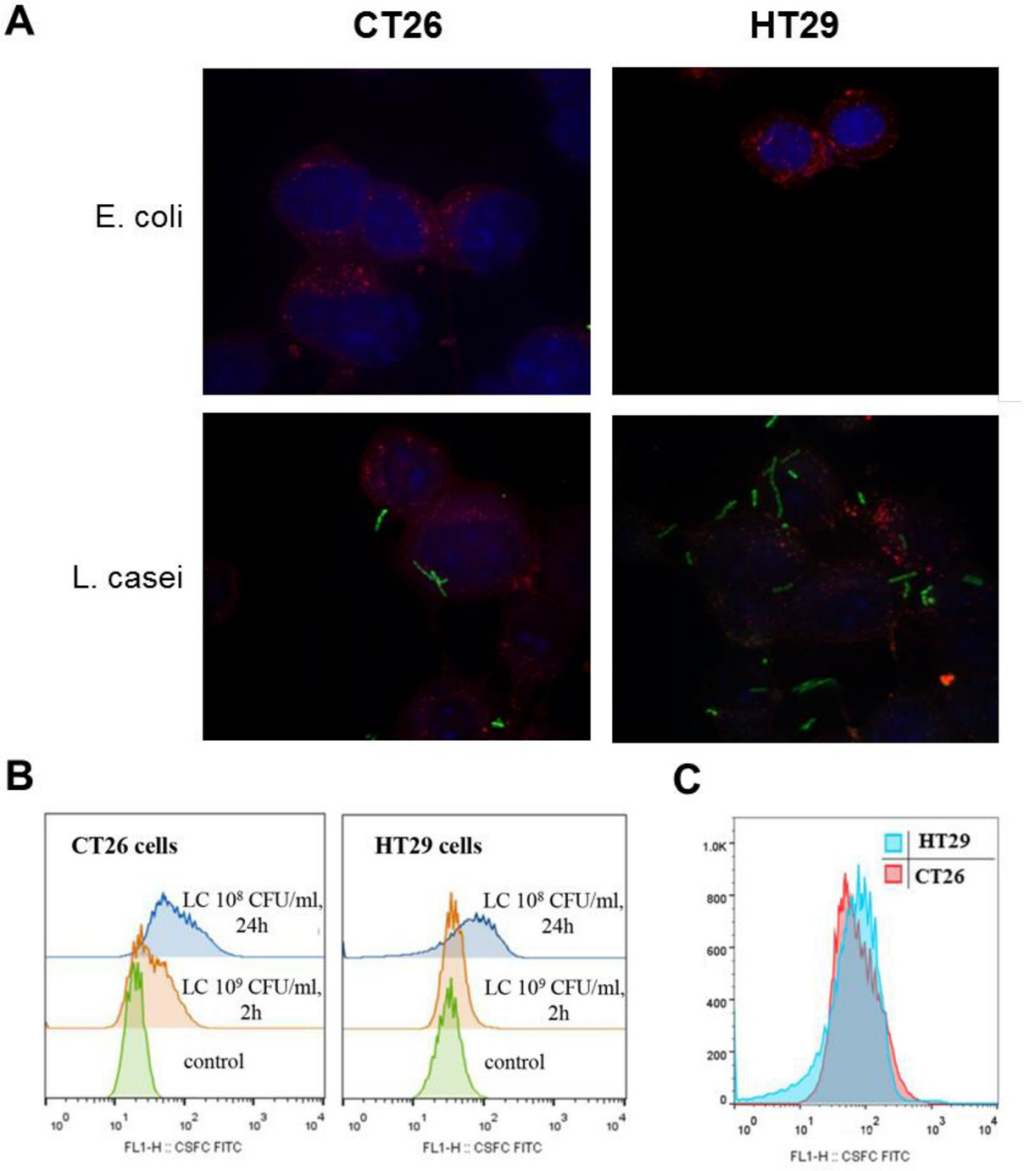
Fluorescently-labeled live *Lactobacillus casei* adheres to human and murine colon cancer cells. (A) Live *L*. *casei* or *E*. *coli* were stained with 20 μM CFSE. Live CT26 or HT29 cells were stained with the nuclear dye Hoechst 33342 and the cytoplasmic membrane dye CellBrite Red dye, and then treated with 10^9^ CFU/mL *L*. *casei* or *E*. *coli* for 5 h and observed by confocal fluorescence microscopy (Stack of 2D images, Magnification: 1000x). (B) Flow cytometric analysis of adhesion. The adhesion of CFSE-labeled *L*. *casei* on murine CT26 or human HT29 cells was detected by the shift in FL1 intensity. Cells were co-incubated with 10^9^ CFU/ml CFSE-stained *L*. *casei* for 2 hours or with 10^8^ CFU/mL for 24 hours. At least 50.000 cells per sample were analyzed. CFSE-labeled bacteria in suspension were also analyzed as a background control but due to the size difference were not detected in the flow cytometric settings used. (C) Comparison of fluorescence intensity profiles of CT26 and HT29 cells treated with 10^8^ CFU/mL *L*. *casei* for 24 hours.

*L*. *casei* was examined for its ability to adhere to colon cancer cell lines. In contrast to *E*. *coli*, adherence of *L*. *casei* to CT26 or HT29 cells—using CFSE-stained live bacteria—was visualized by confocal microscopy (Fig 3A) and detected by flow cytometry (Fig 3B). Cancer cells were treated either with 10^8^ CFU/mL live *L*. *casei* for 24 hours, or with 10^9^ CFU/mL bacteria for 2 hours. Flow cytometric analysis was performed considering the CFSE-stained *L*. *casei* population in suspension as background control. A shift in fluorescence intensity (FL1) indicated the presence of attached bacteria to cells (Fig 3C). Adhesion of live fluorescently-labeled bacteria to cells was already observed after 2 hours of co-incubation with colon cancer cells. Fluorescence intensity in both cell lines due to adherent bacteria was statistically higher after 24 hours co-incubation with live *L*. *casei* compared to control cells. Comparison of the fluorescence intensity patterns of CT26 or HT29 cells did not detect major differences in *L*. *casei* adhesion profiles. Thus, the ability of *L*. *casei* to adhere to either CT26 or HT29 cells *in vitro* was time- and concentration-dependent and did not vary considerably (Fig 3C).

### *Lactobacillus casei* promotes apoptotic cell death in colon cancer cells

**Fig 4:**
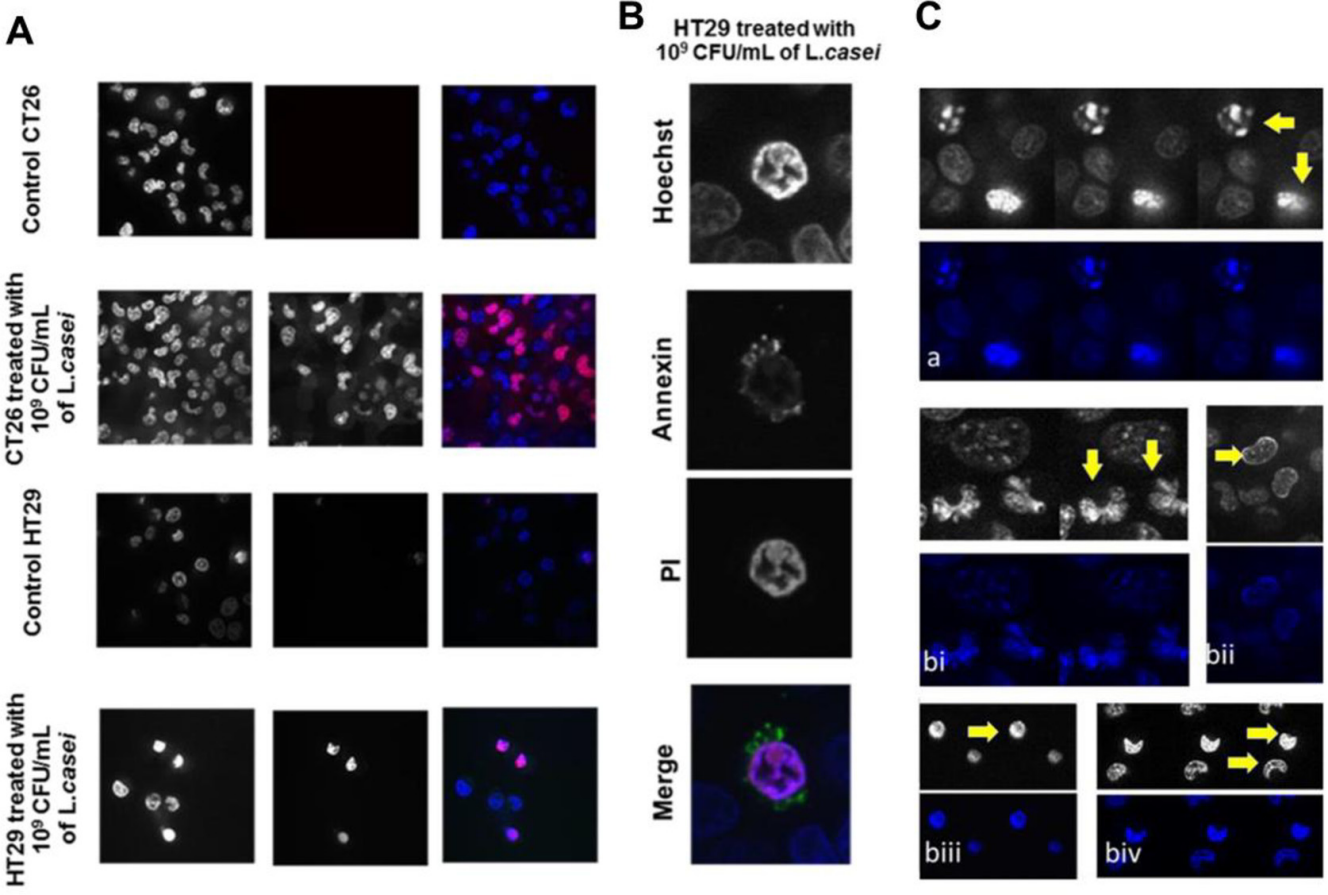
*Lactobacillus casei* induces apoptotic cell death in colon cancer cells detected by confocal microscopy. (A) CT26 or HT29 cells were co-incubated with 10^9^ CFU/mL *L*. *casei* for 24 hours and stained with propidium iodide (PI). Following treatment, live PI-positive CT26 (A) or HT29 (B) cells were visualized (red) as compared to control non-treated cells. Nuclei were counter-stained with Hoechst 33342 (blue) (Magnification: 400x). (B) A representative late apoptotic HT29 cell following *L*. *casei* treatment (Magnification: 800x). HT29 cells were co-incubated with 10^9^ CFU/mL *L*. *casei* for 24 h and stained with Hoechst, PI and Annexin V-FITC. (C) Detection of apoptotic cell death by chromatin condensation and nucleus segmentation. Fluorescence confocal images (black/white and colour) from successive focal planes of apoptotic nuclei of HT29 (a) or CT26 (b) cells following treatment of cells with 10^9^ CFU/mL *L*. *casei* for 24 h. DNA was stained with Hoechst 33342. Apoptotic nuclei with condensed chromatin (top nucleus in (a) and nuclei in (bii), (biv), (biii)) and fragmented nuclei (a) down and both nuclei in (bi)) are visualized (Magnification: 400x).

**Fig 5:**
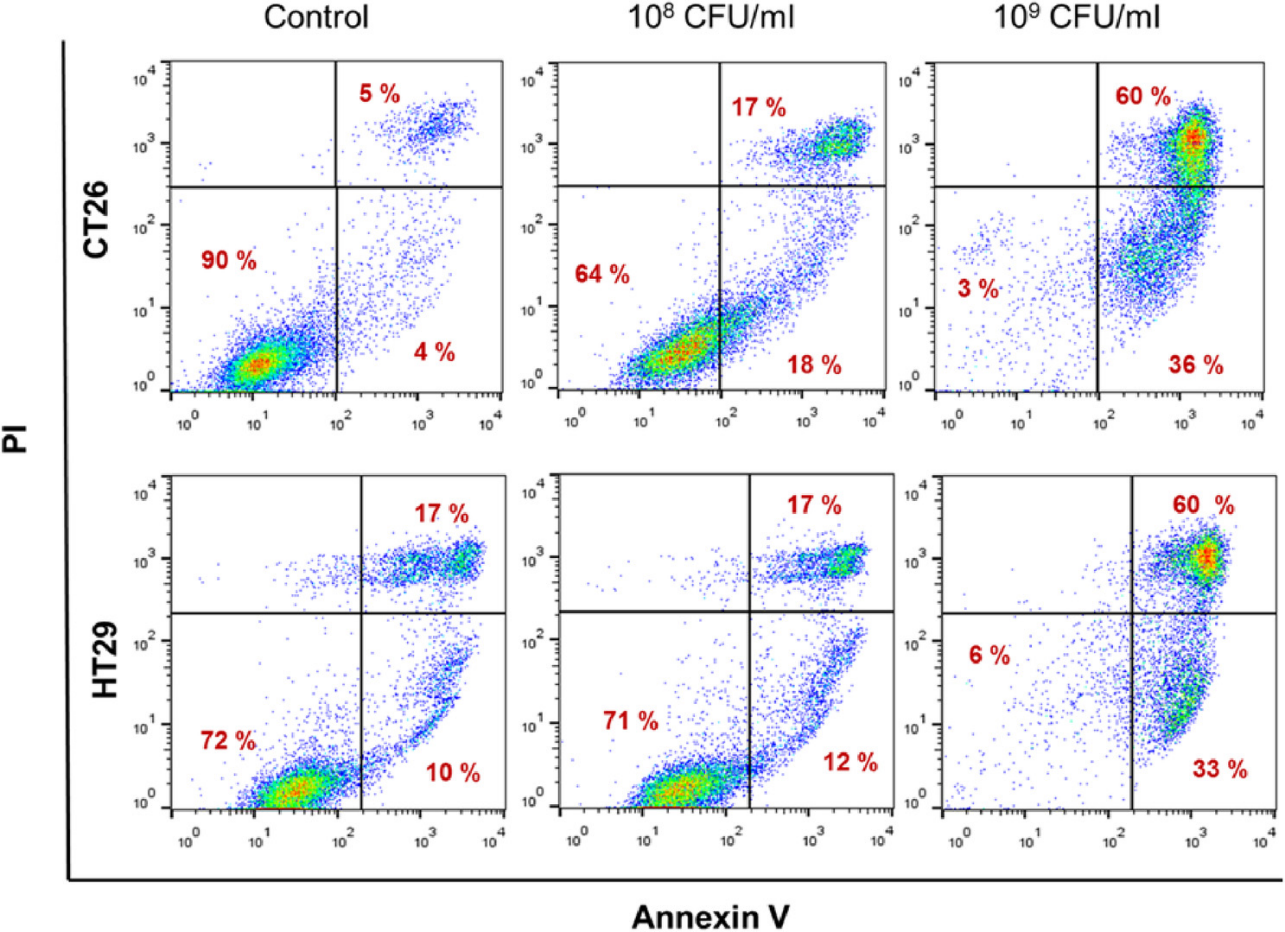
*Lactobacillus casei* induces apoptotic cell death in colon cancer cells detected by flow cytometry. CT26 (upper row) or HT29 cells (lower row) were co-incubated with 10^8^ or 10^9^ CFU/mL *L*. *casei* for 24 hours. Apoptotic cell death of treated cells was detected by dual staining with Annexin V-FITC and PI followed by flow cytometric analysis (see Material and Methods). The percentage of the following cell populations is indicated: Annexin V-FITC and PI negative stained, indicating viable cells (lower left quadrant), Annexin V-positive and PI-negative stained, indicating early apoptotic cells (lower right quadrant), and Annexin V/PI double-stained cells showing late apoptosis (upper right quadrant).

To further investigate the anti-proliferative effects of *L*. *casei*, we examined whether this probiotic strain can promote apoptotic cell death. Cell death was first monitored by live cell imaging using the DNA stain propidium iodide. We observed signs of cell death in colon cancer cells incubated with *L*. *casei*. Specifically, following incubation with 10^8^ or 10^9^ CFU/mL bacteria for 24 hours, CT26 and HT29 cells positive for propidium iodide were detected by confocal microscopy (Fig 4A and 4B). In addition, using the vital dye Hoechst 33342, chromatin condensation and nucleolus segmentation were observed in both cell lines after incubation with live *L*. *casei* for 24 hours (Fig 4C). Apoptotic cell death of colon cancer cells was confirmed by annexin V (FITC) and propidium iodide (PI) double-staining and flow cytometric analysis. Quantification of annexin V and PI positive cells showed that treatment with *L*. *casei* resulted in a concentration-dependent increase in apoptotic (annexin V-positive) colon carcinoma cells (Fig 5). For HT29 cells, the increase in early apoptotic cells raised from 4% (control, non-treated) to 18% (treated with 10^8^ CFU/mL) and 36% (treated with 10^9^ CFU/mL). Co-incubation of CT26 or HT29 cells with 10^9^ CFU/mL live *L*. *casei* for 24 hours showed a significant increase in the percentage of both early and late apoptotic cells as compared to non-treated cells, in fact more than 90% of *L casei*-treated cells (96% annexin-V positive CT26, and 93% annexin V-positive HT29 cells) were apoptotic (Fig 5).

### Live *Lactobacillus casei* upregulates expression of the apoptosis- inducing ligand TRAIL and downregulates transcriptional expression of cyclin D1 and BIRC5a

**Fig 6:**
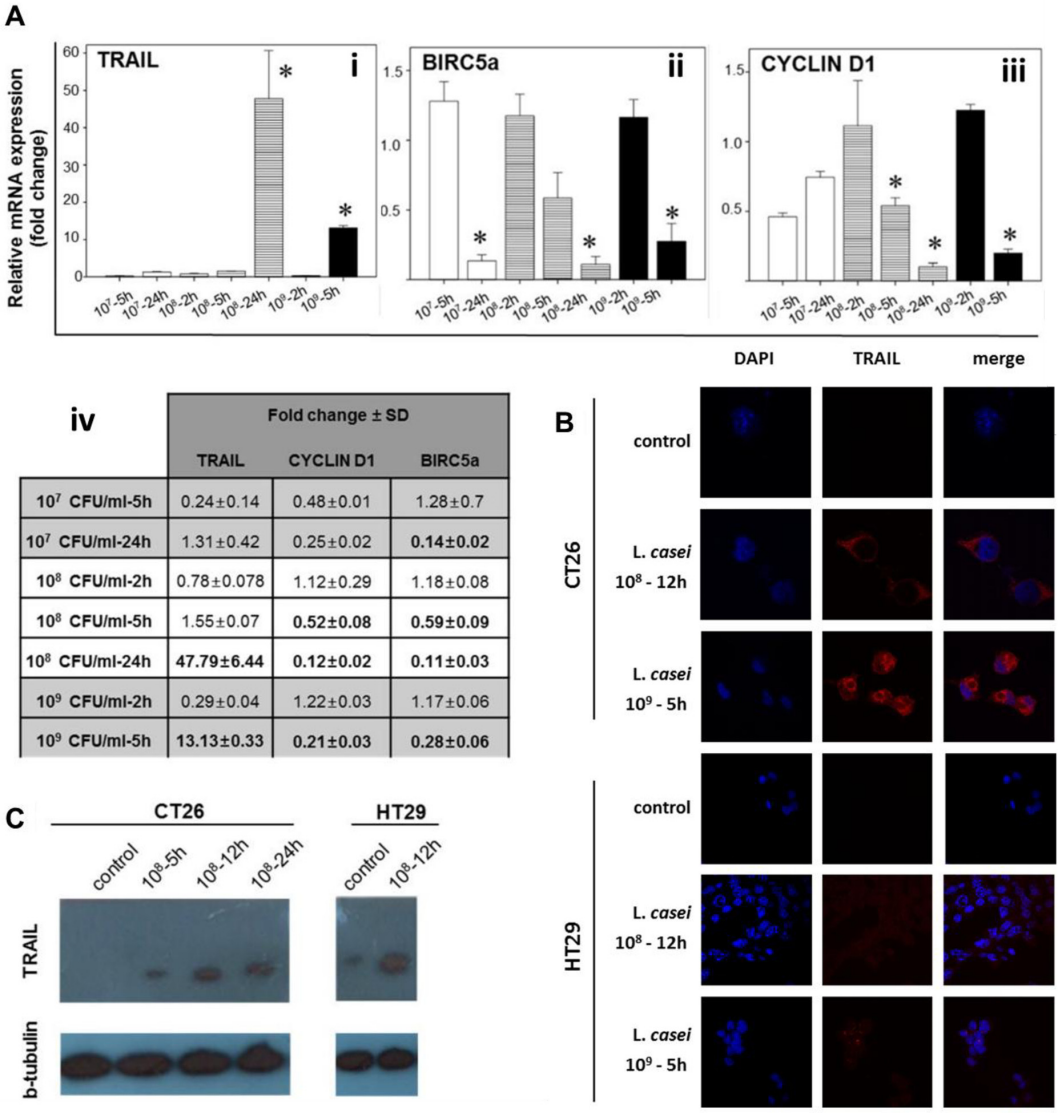
*Lactobacillus casei* upregulates the expression of the apoptosis-inducing ligand TRAIL and downregulates transcriptional expression of cyclin D1 and BIRC5a in colon cancer cells. (A) Relative gene expression (mean fold change) of TRAIL (Ai), BIRC5a (Aii) and cyclin D1 (Aiii) in *L*. *casei*-treated versus non-treated CT26 cells. The values of mean fold change in gene expression are presented in Aiv). CT26 cells were co-incubated with increasing concentrations of *L*. *casei* at indicated timepoints, and then RT-PCR was carried out with specific primers. For analysis, beta-actin was used as the internal reference and non-treated CT26 cells were used as the calibrator. mRNA relative expression for all genes was calculated by the comparative quantification Ct method (ΔΔCt). Results are representative of three independent experiments. Asterisks in (Ai), (Aii) and (Aiii) and numbers in bold in (Aiv) represent statistically significant differences. (B) TRAIL expression is detected by confocal fluorescence microscopy in CT26 or HT29 colon cancer cells after incubation with 10^8^ CFU/mL *L*. *casei* for 12 hours or 10^9^ CFU/ml *L*. *casei* for 5 hours. Cell nuclei were stained with DAPI and TRAIL was detected using a specific antibody and visualized by an AlexaFluor647-conjugated secondary antibody (Stack of 2D images, Magnification: 1000x). (C) TRAIL expression was examined by Western blot analysis in CT26 and HT29 cells. Cells were treated with 10^8^ CFU/mL *L*. *casei* for different time points. Membrane protein extract was used to detect TRAIL protein with a specific antibody against TRAIL and the respective soluble protein fraction was used to determine β-tubulin protein expression with a specific anti-β-tubulin antibody. Left gel, control non-treated CT26 cells (lane 1), CT26 cells treated with 10^8^ CFU/mL *L*. *casei* for: 5 hours (lane 2); 12 hours (lane 3); 24 hours (lane 4). Right gel, non-treated HT29 cells (lane 1); HT29 cells treated with 10^8^ CFU/mL *L*. *casei* for 12 hours (lane 2).

In order to investigate the molecular events leading to the apoptotic effect, we examined the expression of apoptosis-related genes. The comparative differential expression of selected genes was examined by SYBR-Green Real-time PCR in *L*. *casei*-treated CT26 cells compared to non-treated cells. Transcription levels of each gene were normalized with beta-actin Ct values. CT26 colon cancer cells were treated with increasing concentrations of live *L*. *casei* for different timepoints. Following treatment with 10^9^ or 10^8^ CFU/mL *L*. *casei*, a statistically higher gene expression of TRAIL was observed at 5- and 24- hours post-treatment, respectively (Fig 6Ai). Co-incubation of CT26 cells with 10^8^ CFU/mL *L*. *casei* for 24 hours resulted in approximately 60 fold higher mRNA expression of TRAIL as compared to control non-treated cells or cells treated for 2 hours only. For the same time period and concentrations, BIRC5a mRNA, encoding for the anti-apoptotic protein Survivin, was significantly downregulated (Fig 6Aii). Apparently, even co-incubation with 10^7^ CFU/mL live bacteria induced significant transcriptional down-regulation of BIRC5a mRNA at 24 hours post-treatment. For cyclin D1, a transcriptional down-regulation was observed in a time-dependent manner following treatment of colon cancer cells with 10^8^ or 10^9^ CFU/mL bacteria. Treatment of CT26 cells for 24 h with 10^8^ CFU/ml *L*. *casei* resulted in an approximately 10 fold lower mRNA expression of cyclin D1 and BIRC5a, as compared to control non-treated cells or cells treated for 2 hours. Significant down-regulation of cyclin D1 mRNA was detected after 5 hours co-incubation of CT26 cells with 10^8^ and 10^9^ CFU/mL *L*. *casei* (Fig 6Aiii). The mean fold change in transcriptional gene expression for TRAIL, cyclin D1 and BIRC5a in *L*. *casei*-treated versus non-treated cells is presented in Fig 6Aiv. Noteworthy, by immunofluorescence analysis, increased expression of TRAIL at protein level was observed in CT26 or HT29 cells after co-incubation with 10^8^ CFU/mL bacteria for 12 hours or 10^9^ CFU/mL for 5 hours (Fig 6B). Moreover, increased expression of TRAIL was detected by Western blot analysis in both CT26 and HT29 cells incubated with 10^8^ CFU/mL *L*. *casei* (Fig 6C). Thus, up-regulation of TRAIL mRNA and protein as well as down-regulation of cyclin D1 and BIRC5a mRNA corroborated the growth-inhibitory effect.

### Oral administration of live *Lactobacillus casei* inhibits *in vivo* growth of colon carcinoma in mice

**Fig 7:**
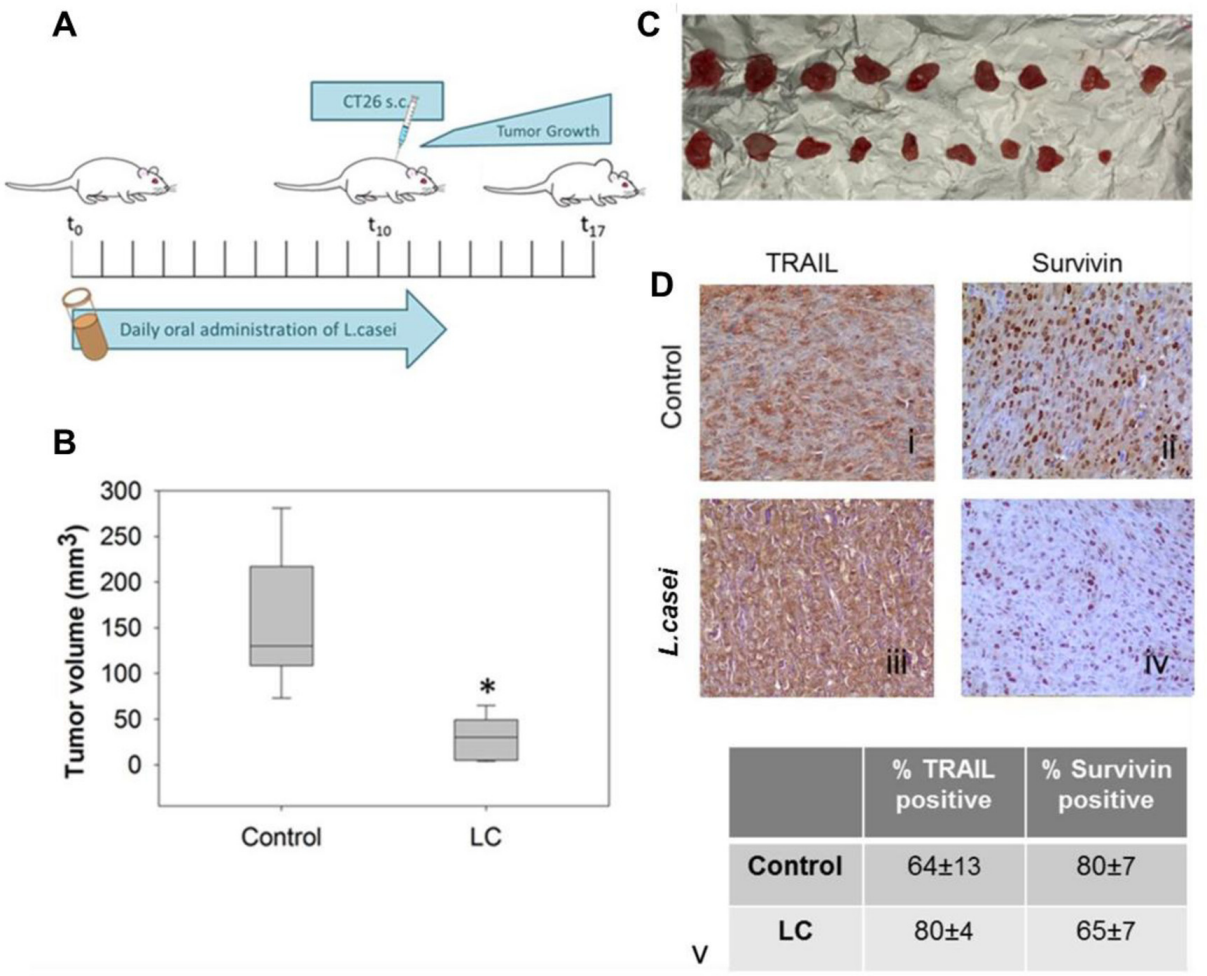
Oral administration of live *Lactobacillus casei* inhibits *in vivo* growth of colon carcinoma tumors in mice. (A) Schematic representation of the *in vivo* tumor model. Live *L*. *casei* was administered *per os* daily to BALB/c mice for 13 days. At day 10, 5x10^6^ CT26 cells were inoculated subcutaneously. Mice in the control group received PBS. Tumors were harvested from euthanized animals 7 days after administration of CT26 cells. (B) Mean tumor volume of tumors excised from mice that received *L*. *casei* (LC) or PBS (control). A statistically significant (*p* < 0.001) reduction of ≈80% in tumor volume was observed in *L*. *casei*-treated mice as compared to control. (C) Photographic observation of tumors harvested from control (PBS)- or *L*. *casei* (LC)-treated mice. (D) Immunohistochemical detection of TRAIL (Di, Diii) and Survivin (Dii, Div) in CT26 tumors in BALB/c mice. Detection of TRAIL in tumor tissue from *L*. *casei*-treated (Diii) compared to control (Di) mice, and Survivin in tumor tissue from *L*. *casei*-treated (Div) or control (Dii) mice. At least ten sections per tumor and seven tumors per group from two independent experiments were analyzed. The difference in the expression of TRAIL in tumor tissue from treated (Diii) versus control (Di) mice is statistically significant (*p* = 0.007), as calculated using the SigmaPlot 11.0 statistical software.

Notably, the protective anti-tumor effect of live *L*. *casei* was confirmed in a murine colon carcinoma model. 10^9^ CFU live bacteria were daily administered *per os* to BALB/c mice for 13 days. Control mice received an equal volume of PBS. At day 10, 5x10^6^ proliferating CT26 cells were inoculated subcutaneously. Seven days later, euthanasia was carried out by cervical dislocation and tumors, as well as other organs, were harvested (Fig 7A). During the experimental procedure no signs of disease or discomfort were observed in both groups of mice. Box-plots in Fig 7B present the mean tumor volume of each group. The mean tumor volume of *L*. *casei*-treated BALB/c mice was significantly reduced compared to control mice (*p* <0.001). Tumor volume inhibition was 81.7% for *L*. *casei*-treated mice as compared to control ones. This finding was reproduced in another independent experiment and tumor volume inhibition reached 85%. As illustrated in Fig 7C, in the control group all mice developed tumors, while only 90% of *L*. *casei*-treated mice developed tumors of reduced volume. Thus, oral administration of this probiotic strain in mice resulted in the formation of tumors with reduced tumor volume. In particular, a statistically significant tumor volume inhibition was evident in *L*. *casei*-treated mice as compared to control ones. Moreover, protein expression of TRAIL and Survivin was examined in colon carcinoma murine tumor tissues by immunohistochemical analysis with specific antibodies. Interestingly, and in support of the *in vitro* data, tumors from *L*. *casei*-treated mice showed higher expression of TRAIL and lower expression of Survivin than tumors from control mice (Fig 7D).

## Discussion

*Lactobacillus casei* ATCC 393 is a nonpathogenic facultatively anaerobic bacterium and probiotic strain used in fermented dairy products and functional foods [40–43]. Here, we investigated and provide evidence for anti-proliferative and pro-apoptotic effects of *L*. *casei* ATCC 393 that mediate anti-neoplastic effects against colon carcinoma *in vitro* and *in vivo*. In a similar study, Chen *et al*. [23] demonstrated that oral pre-inoculation of mice with *L*. *acidophilus* NCFM for 14 days reduced CT26 tumor volume by 50% compared to control mice. The authors reported down-regulation of expression of the surface proteins CXCR4 and MHC class I in the colon, mesenteric lymph nodes and spleen tissue of BALB/c mice, and suggested that these changes may be associated with *L*. *acidophilus*-induced modulation of the host’s immune response [23]. It is generally believed that specific LAB strains can beneficially activate anticancer mechanisms, thereby regulating host’s immune response [6,26,53]. Probiotic actions such as adhesion of LAB or their components to epithelial cells as well as release of soluble factors have been proposed to be important for the suppression of neoplastic cells [13]. In our experiments, live bacteria induced potent anti-proliferative effects to colon cancer cells. The proposed effects may be mediated at least in part due to soluble factors, for example bioactive metabolites, which are secreted from live bacteria. Apparently, cell-free supernatants exerted anti-proliferative effects in a time- and concentration-dependent manner. Compared to live bacteria, high concentrations of cell-free supernatants were needed to induce a considerable growth inhibition in colon cancer cells. It may well be that live bacteria have a higher impact than CFS, since they continuously produce active soluble metabolites, postbiotics, which are able to mediate sustained growth-inhibitory effects towards tumor cells. Moreover, certain bioactive metabolites may induce beneficial immune responses and signaling pathways *in vivo*, thereby reducing the viability and progression of colon cancer cells, indirectly. In a recent study, cell-free supernatants (CFS) from two LAB strains, *L*. *casei* and *L*. *rhamnosus* GG, were shown to inhibit colon cancer cell invasion by influencing matrix metalloproteinase-9 (MMP-9) activity and levels of the tight junction protein zona occludens-1 (ZO-1) in cultured metastatic human colorectal carcinoma cells [54]. In another study, *L*. *gasseri* SF1183 produced molecule(s) able to interfere drastically with HCT116 cell proliferation and stimulate G1-phase arrest [55]. Notably, LAB do not exclusively act in the large intestine by affecting intestinal microbiota but also mediate some of the mechanisms (immunological modulation or provision of bioactive metabolites) in other organs. In particular, some of these interactions and beneficial effects may be mediated or influenced by the *in situ* production of SCFA. It has been reported that acidic extracellular pH shifts colorectal cancer cell death from apoptosis to necrosis (below pH 6.0) upon exposure of HT-29 cells to SCFA from probiotic propionibacteria [56].

In the present study live *L*. *casei* ATCC393 adhered to colon cancer cells, reduced cell viability in a time- and concentration-dependent manner and induced apoptotic cell death. Apoptosis is a natural physiological process of programmed cell death that regulates homeostasis and represents an ideal target for anti-neoplastic strategies. Through a series of regulated processes, cancer cells become fragmented and their residual portions are absorbed by adjacent tissues and immune cells [57]. Several studies showed that LAB can play a role in the regulation of apoptosis via intrinsic and extrinsic pathways that are potentially critical mechanisms in the prevention of colorectal cancer, as reviewed by Zhong *et al*. [5]. Oral administration of *Lactobacillus acidophilus* in mice reduced the severity of colorectal cancer and enhanced apoptosis in treated mice [23]. *Lactobacillus reuteri* was shown to prevent colorectal cancer via downregulating NF-kB-dependent gene products that regulate cell proliferation (COX-2, cyclin D1) and survival (Bcl-2, Bcl-xL) [58]. Other studies reported that exopolysaccharides of *L*. *acidophilus* and *L*. *rhamnosus* were anti-tumorigenic against HT29 colon cancer cells and that this activity was due to activation of autophagic cell death [24]. Moreover, some strains of LAB can enhance apoptosis-inducing capacity of chemotherapeutic agents [59]. In addition, apoptosis induction could represent a mechanism by which commensal and/or probiotic bacteria could prevent proliferation of dysplastic cells. It was proposed to represent a physiologic "oncologic surveillance" mechanism for colonic proliferative disease prevention [3,60].

In the *in vitro* studies, *L*. *casei* downregulated in a time-dependent manner the transcriptional expression of CCND1 coding for cyclin D1 (a protein required for progression through the G1 phase of cell cycle) and BIRC5a encoding the anti-apoptotic protein Survivin (negative regulator of apoptosis, expressed highly in most human tumors). Treatment of CT26 cells for 24 h with 10^8^ CFU/mL *L*. *casei* resulted in approximately 10 fold lower mRNA expression of cyclin D1 and BIRC5a as compared to control non-treated cells or cells treated for a period of 2 hours. Notably, down-regulation of Survivin/BIRC5a expression was also detected in tumor tissues of *L*. *casei*-treated mice compared to tumor tissues from control mice. Thus, *L*. *casei* inhibited growth and induced apoptotic cell death in colon carcinoma cells, perhaps through modulating the expression of genes involved in cell cycle progression and tumor growth.

In addition, *L*. *casei* induced a strong dose- and time-dependent transcriptional expression of TRAIL in colon carcinoma cells. TRAIL up-regulation was also detected at protein level in *L*. *casei*-treated CT26 or HT29 cells. Similarly, TRAIL protein expression was higher in tumors from *L*. *casei*-treated mice as compared to tumors from control mice. Conceivably, TRAIL over-expression may be involved in the apoptotic process initiated by *L*. *casei* treatment. TRAIL has been shown to selectively induce apoptosis in many tumor cell lines without affecting normal cells and tissues, and has been regarded as a promising therapeutic agent against colorectal malignancies [61]. Upon binding to its death receptors (DR4 and DR5), TRAIL activates the extrinsic apoptosis signaling pathways, it regulates caspase 8 that triggers a signaling cascade that involves Bid, Bax, Bcl-2 and other caspases, such as 9 and 3. It can also be expressed by various cells of the immune system, depending on their status of stimulation [62]. TRAIL was previously reported to play an important role in LAB-induced anti-neoplastic effects, since *L*. *plantarum* enhanced the natural killer (NK) activity through endogenous TRAIL production. In this study, TRAIL was shown to be the key molecule in causing cytotoxicity against malignant cells induced by *L*. *plantarum* [63]. In particular, soluble TRAIL alone or in combination with other molecules and factors (such as actinomycin D) has been documented to sensitize colon carcinoma cells to apoptotic cell death [64–65]. Interestingly, in a number of recent studies pharmacologic agents or bioactive compounds sensitized colon carcinoma cells towards TRAIL-induced cytotoxicity via down-regulation of Survivin expression. In these studies resistance of colon cancer cells to (apoptotic) cell death could be overcome through inhibition of Survivin expression [66–68]. Noteworthy, TRAIL not only functions to induce cell apoptosis, but also works as a key effector molecule in the immune system [62]. Indeed, TRAIL can also induce tumor inhibitory effects by regulating host immune responses. In a recent study, TRAIL suppressed hepatocellular tumor growth in mice by inducing specifically tumor-infiltrating CD4^+^ CD25^+^ Treg apoptosis [69], thus, leading to suppression of adverse immune responses. Thus, TRAIL may either directly induce apoptotic cell death in colon carcinoma cells and/or indirectly, through regulation of immune responses. Further studies are needed to evaluate the role of TRAIL as a potential novel cytotoxicity mechanism in *L*. *casei*-induced tumor growth inhibition.

Although, the underlying mechanisms deserve further analysis, our data show clearly that intact live *Lactobacillus casei* ATCC393 exerts potent anti-proliferative, growth-inhibitory and pro-apoptotic effects *in vitro*. The results were further confirmed *in vivo* as oral administration of *L*. *casei* significantly impaired tumor growth in an experimental *in vivo* colon carcinoma model, suggesting that the use of this probiotic strain in dietary intervention programs and functional foods may hold promise. Inhibition of *in vivo* tumor growth mediated by this probiotic strain may be induced through a combination of *direct* effects (mediated by LAB components and/or LAB-secreted soluble factors) and *indirect* effects through modulation of effective immune responses responsible for inhibiting growth of malignant cells present at extra-intestinal sites.

## Abbreviations

BCA: bicinchoninic acid
BIRC5: baculoviral IAP repeat containing 5
BSA: bovine serum albumin
CFS: cell-free supernatant
CFSE: carboxyfluorescein succinimidyl ester
CFU: colony-forming units
DMEM: Dulbecco's modified Eagle's medium
DMSO: dimethylsulfoxide
EPR: enhanced permeability and retention
FITC: fluorescein isothiocyanate
HEPES: 4-(2-hydroxyethyl)-1-piperazineethanesulfonic acid
HK-SON: heat-killed and sonicated
HPLC: high performance liquid chromatography
LAB: lactic acid bacteria
MMP-9: matrix metalloproteinase 9
MRS medium: de Man, Rogosa and Sharpe medium
NF-κB: nuclear factor kappa B
NK: natural killer cells
PBS: phosphate buffered saline
PI: propidium iodide
SCFA: short-chain fatty acids
SRB: sulforhodamine B
TRAIL: Tumor necrosis factor-related apoptosis-inducing ligand
